# Whey Protein Sodium-Caseinate as a Deliverable Vector for EGCG: In Vitro Optimization of Its Bioaccessibility, Bioavailability, and Bioactivity Mode of Actions

**DOI:** 10.3390/molecules29112588

**Published:** 2024-05-31

**Authors:** Ali Korin, Mostafa M. Gouda, Mahmoud Youssef, Eman Elsharkawy, Amgad Albahi, Fuchao Zhan, Remah Sobhy, Bin Li

**Affiliations:** 1 College of Food Science and Technology, Huazhong Agricultural University, Wuhan 430070, China; 2 Food Science and Technology Department, Faculty of Agriculture, Al-Azhar University, Cairo 11651, Egypt; 3 College of Biosystems Engineering and Food Science, Zhejiang University, Hangzhou 310058, China; 4 Department of Nutrition & Food Science, National Research Centre, Dokki, Giza 12622, Egypt; 5 Faculty of Science, Northern Border University, Arar 91431, Saudi Arabia; 6 National Food Research Centre, Ministry of Agriculture and Natural Resources, Khartoum 113, Sudan; 7 Department of Biochemistry, Faculty of Agriculture, Benha University, Moshtohor 13736, Egypt

**Keywords:** epigallocatechin gallate, polyphenol, bioaccessibility, molecular docking, Caco-2 cell assay, anti-proliferative activity

## Abstract

Epigallocatechin gallate (EGCG), the principal catechin in green tea, exhibits diverse therapeutic properties. However, its clinical efficacy is hindered by poor stability and low bioavailability. This study investigated solid particle-in-oil-in-water (S/O/W) emulsions stabilized by whey protein isolate (WPI) and sodium caseinate (NaCas) as carriers to enhance the bioavailability and intestinal absorption of EGCG. Molecular docking revealed binding interactions between EGCG and these macromolecules. The WPI- and NaCas-stabilized emulsions exhibited high encapsulation efficiencies (>80%) and significantly enhanced the bioaccessibility of EGCG by 64% compared to free EGCG after simulated gastrointestinal digestion. Notably, the NaCas emulsion facilitated higher intestinal permeability of EGCG across Caco-2 monolayers, attributed to the strong intermolecular interactions between caseins and EGCG. Furthermore, the emulsions protected Caco-2 cells against oxidative stress by suppressing intracellular reactive oxygen species generation. These findings demonstrate the potential of WPI- and NaCas-stabilized emulsions as effective delivery systems to improve the bioavailability, stability, and bioactivity of polyphenols like EGCG, enabling their applications in functional foods and nutraceuticals.

## 1. Introduction

Green tea (*Camellia sinensis*) contains several water-soluble catechins, with epigallocatechin gallate (EGCG) being the most abundant and biologically active polyphenolic compound [1]. EGCG has potentially extensive therapeutic effects in diseases associated with increased reactive oxygen species (ROS) and oxidative stress, such as cancer and cardiovascular diseases [2,3], as well as their modulation of molecular signaling pathways, enzyme activities, and interactions with membrane receptors implicated in cognitive functioning and Alzheimer’s disease [4]. Numerous studies in humans and animals have demonstrated that EGCG exhibits diverse therapeutic properties including immunomodulatory, cardioprotective, anti-tumor, hypoglycemic, and neuroprotective effects [5,6,7,8]. However, the clinical efficacy of EGCG is hampered by its poor stability and low bioavailability in vivo [9,10]. EGCG is highly prone to degradation under the alkaline conditions of the small intestine and in the presence of digestive enzymes, resulting in reduced intestinal absorption [9]. Overcoming the limitations of EGCG stability and bioavailability is, thus, critical to fully realizing its therapeutic potential. 

Motivated by such benefits, great efforts were made to enhance the stability and bioavailability of EGCG. For instance, novel delivery systems, such as emulsions, may improve EGCG absorption by providing protection from the harsh gastro-intestinal tract environment [11]. Meanwhile, further research is necessary to develop and optimize delivery systems that enhance EGCG bioavailability. As a result, recent studies were focused on formulation strategies to improve EGCG stability and bioavailability [12]. Milk proteins, including whey protein isolate-sodium caseinate (WPI-NaCas), have shown promise as emulsion stabilizers. Whey protein isolate (WPI) constitutes around 20% of bovine milk proteins, while caseins represent 80% [13]. Whey proteins have been recognized as potential as emulsifiers and carriers of bioactive compounds [14,15]. Caseins also possess binding capacities for polyphenols like EGCG [16]. Therefore, in some situations, employing WPI-NaCas may improve EGCG’s bioaccessibility. Before investing in expensive in vivo trials, in vitro digestion and Caco-2 absorption models offer helpful preliminary methods to screen delivery systems by calculating bioaccessibility and intestinal transport [17]. The solid-in-oil-in-water (S/O/W) technique dissolves a solid drug or bioactive compounds in oil and then disperses the solid–oil suspension in water [18]. S/O/W emulsions overcome the limitations of conventional W/O/W emulsions for hydrophilic drugs, providing improved stability, encapsulation efficiency, and release properties [19]. Solid-state encapsulation enhances protein stability compared to aqueous solutions [20]. The S/O/W method enables effective encapsulation of sensitive materials [21]. The potential regulatory implications of using WPI- and NaCas-stabilized emulsions in functional foods and pharmaceuticals are significant. These emulsions can enhance the stability, bioaccessibility, and bioavailability of bioactive compounds like EGCG, making them promising delivery systems for health-promoting ingredients [10]. However, the successful application of these emulsions in food products requires careful consideration of regulatory aspects, such as safety evaluation and compliance with food additive regulations [12]. This was studied by assessing the effect of the emulsions on Caco-2 cell viability, which provides insight into their potential impact on intestinal health and barrier function. Furthermore, the sensory attributes and consumer acceptance of the final products containing these emulsions need to be assessed to ensure their commercial viability [22].

We hypothesized that solid particle-in-water (S/O/W) emulsions stabilized by WPI and NaCas could serve as effective carriers to improve the bioavailability and intestinal absorption of the green tea polyphenol EGCG. Thus, we aimed to optimize the in vitro delivery of EGCG using WPI-NaCas stabilized emulsions. The anti-proliferative activity of EGCG against cancer cells was utilized to estimate the bioaccessible fraction released from emulsions after in vitro digestion. Caco-2 colorectal cancer cell viability was measured after treatment with digested and undigested EGCG-loaded emulsions to calculate intact EGCG levels. Caco-2 monolayers were also implemented to evaluate EGCG transport from emulsions versus free solutions. Furthermore, the emulsion effects on Caco-2 viability and oxidative stress protection were explored. Lib-dock-based molecular docking was also used to understand the protection effects of both WPI and NaCas on EGCG. This study provides useful data on the potential of WPI- and NaCas-stabilized emulsions to augment EGCG bioaccessibility, stability, absorption, and bioactivity using in vitro models before costly animal and human trials. Findings could help optimize emulsion-based delivery systems to improve EGCG bioavailability. Ultimately, enhancing the delivery of EGCG and polyphenols has important implications for the development of emulsions as functional foods and nutraceuticals.

## 2. Results and Discussion

### 2.1. Encapsulation Efficiency and Retention Efficiency

**Figure 1:**
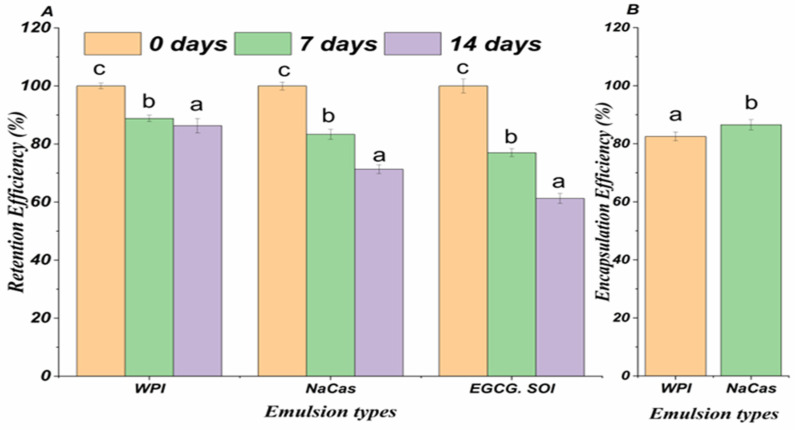
(**A**) Encapsulation efficiency of EGCG. (**B**) The RE of EGCG of the emulsions during different storage periods. Error bars signify standard error of the mean. Different letters are statistically significant at *p* < 0.05.

The encapsulation efficiency (EE) and retention efficiency (RE) of EGCG in whey protein isolate (WPI)- and sodium caseinate (NaCas)-stabilized emulsions were investigated to evaluate their potential as delivery systems (Figure 1A,B). The EE values were 82.52 ± 1.54 and 86.53 ± 1.78% for WPI- and NaCas-stabilized emulsions, respectively, indicating a successful incorporation of EGCG within the emulsion matrix.

The RE of EGCG in the emulsions was also assessed during storage at 4 °C for 14 d. After 7 days, the RE values were 90.24 ± 2.12 and 92.37 ± 1.89% for WPI- and NaCas-stabilized emulsions, respectively. At the end of the 14-d storage period, the RE values remained high, with 87.19 ± 2.45 for WPI-stabilized and 89.76 ± 2.08% for NaCas-stabilized emulsions. The high EE and RE values observed for EGCG in WPI-bioactive compounds emulsions demonstrate their effectiveness in encapsulating and retaining the bioactive compounds. The emulsion matrix likely provides a protective barrier against environmental factors that can degrade EGCG, such as oxidation and pH changes [23]. The slightly higher EE and RE values observed for NaCas-stabilized emulsions compared to WPI-stabilized emulsions may be attributed to the greater surface activity and emulsifying properties of caseins [24]. The superior performance of NaCas-stabilized emulsions over WPI-stabilized emulsions can be attributed to the unique structural and functional properties of caseins. Caseins possess a flexible, disordered structure with distinct hydrophobic and hydrophilic regions, enabling them to rapidly adsorb and form a cohesive interfacial layer around the oil droplets [25]. The high degree of hydrophobicity in caseins facilitates their anchoring at the oil–water interface, while their flexible structure allows for efficient rearrangement and close packing at the interface, resulting in a thicker and more stable interfacial film [26]. Moreover, NaCas exhibits excellent emulsifying capacity due to its ability to effectively suspend and stabilize oil droplets within the aqueous phase. The charged and amphiphilic nature of caseins contributes to enhanced electrostatic and steric stabilization of the emulsion droplets, preventing coalescence, and promoting long-term stability [27]. The ability of these milk protein-stabilized emulsions to maintain high EGCG retention during storage suggests their potential for prolonged shelf life and sustained delivery of the bioactive compounds [28].

### 2.2. Molecular Docking of EGCG with β-Lactoglobulin and NaCas

**Figure 2:**
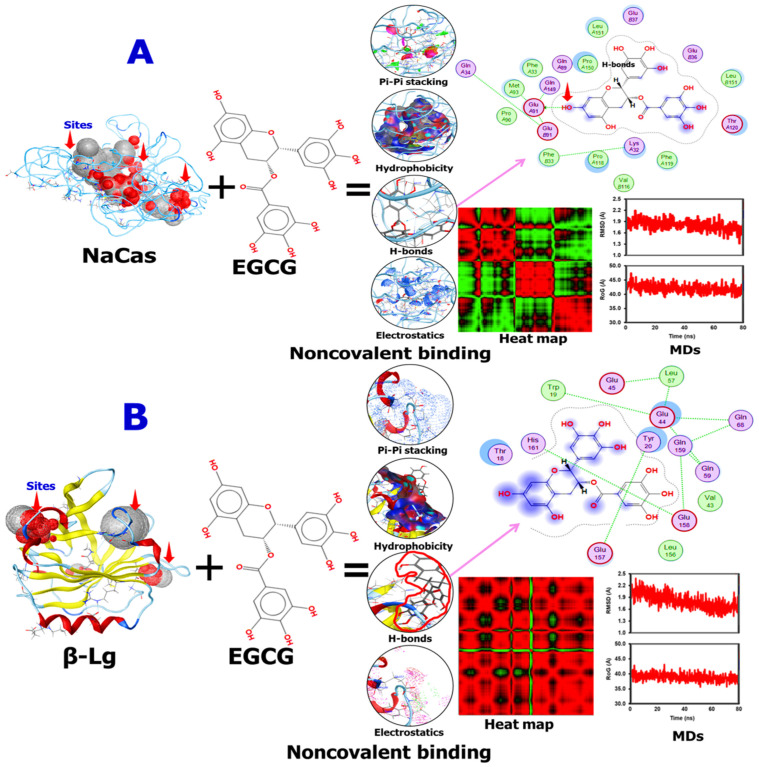
Schematic diagrams showing the interactions of epigallocatechin gallate (EGCG) with sodium caseinate (NaCas) (**A**) or beta-lactoglobulin (β-Lg) (**B**), generated using 2D, 3D, and surface-based diagram features in Accelrys Discovery Studio software V.24.1.0.23298. MDs and heat map were also presented. The Violet arrow is indicating the H-bonds between β-Lg or NaCas and EGCG. Docking parameters were supplemented in Table S1.

The computational results were subsequently utilized to elucidate the binding sites and forces governing the interactions between EGCG and the proteins WPI and NaCas. The three-dimensional diagrams in Figure 2A,B revealed that EGCG interacted with the subunits of NaCas via a single hydrogen bond. Notably, 19 amino acid residues were implicated in the NaCas–EGCG interaction, indicating a stable binding interaction (Figure 2A). More specifically, the hydroxyl group present in the phenolic rings of EGCG formed hydrogen bonds with the GluA91 residue of the NaCas subunits. Additionally, π–π interactions and hydrophobic forces were observed between EGCG and certain amino acids of NaCas. The amino acids involved in these interactions were Thr, Leu, Glu, Leu, Pro, Gln, Phe, Met, Lys, Val, and Thr. Figure 2B portrayed that β-Lg also could interact with EGCG, where 14 amino acids were involved in this interaction, namely Gln, Glu, Tyr, Leu, Trp, His, Thr, Val, and Leu. Most importantly, no direct interaction was noted between β-Lg and EGCG, which might explain their low encapsulation efficiency compared with NaCas which directly interacted with EGCG and protected it. Meanwhile, similar binding forces were found between the two carriers used toward EGCG. The MDs analysis showed that the noncovalent binding between EGCG and NaCas or β-Lg is stable during the run time, representing their stability after digestion and delivery. However, the heat map analysis showed the difference of the EGCG/β-Lg and EGCG/NaCas conjugates, where the late showed more distribution of the amnio acids involved in their interaction compared with the first one, again confirming the stability of EGCG/NaCas conjugate than EGCG/β-Lg one. The molecular docking and dynamics simulation results provide valuable insights into the binding interactions between the milk proteins (β-lactoglobulin and sodium caseinate) and epigallocatechin gallate (EGCG), supporting the claims about the stability and encapsulation capabilities of these delivery systems. As shown in Table 1, the molecular docking simulations predicted favorable binding energies of −5.035 kcal/mol for the β–Lg–EGCG complex and −5.979 kcal/mol for the sodium caseinate-EGCG complex. These negative binding energy values indicate spontaneous and stable binding interactions between the proteins and EGCG. These hydrogen bonding interactions contribute to the stability of the protein-EGCG complexes and may play a role in protecting EGCG from degradation during digestion and delivery.

The root mean square deviation (RMSD) is essential for evaluating protein structural stability, indicating the average deviation from the initial conformation over time. As illustrated in Figure 2, the RMSD of β-lactoglobulin and sodium caseinate (NaCas) complexed with EGCG was compared. The RMSD for β-lactoglobulin fluctuated around 1.90 nm, averaging 1.6 nm, which is significantly lower than the 2.2 nm RMSD observed for the NaCas–EGCG complex at site 1 [29]. This indicates that EGCG binding to site 2 of β-lactoglobulin under normal pressure conditions results in a more stable protein structure compared to the NaCas–EGCG complex. However, the instability of the NaCas–EGCG complex was reduced under high-pressure conditions. These findings align with the observations of Vanaei et al. of increased RMSD when safranal binds to β-lactoglobulin. The results support the hypothesis that the hydroxyl groups in EGCG can form hydrogen bonds with milk protein surface residues, corroborating earlier experimental data [30].

### 2.3. Bioaccessibility Assessment

**Figure 3:**
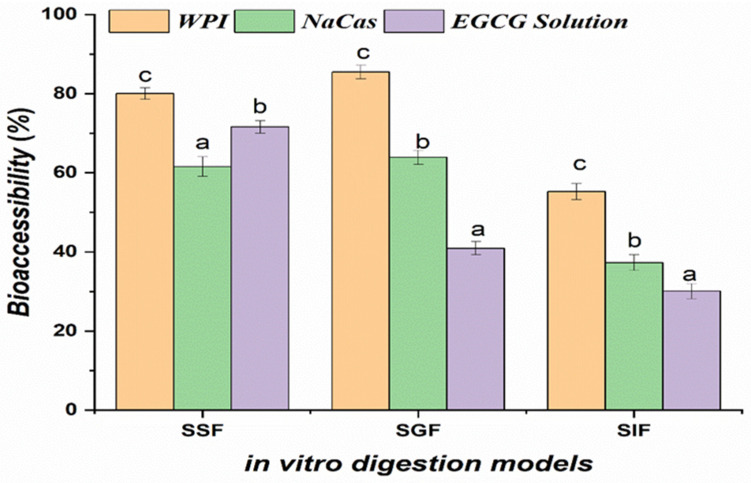
Bioaccessibility of epigallocatechin gallate (EGCG) incorporated in whey protein isolate (WPI) and sodium caseinate (NaCas) emulsions compared to unencapsulated EGCG solutions after simulated gastrointestinal digestion. Error bars signify standard error of the mean. Different letters are statistically significant at *p* < 0.05.

Following the oral phase, the EGCG bioaccessibility values for WPI- and NaCas-stabilized emulsions were 35.12 ± 2.17% and 37.28 ± 1.94%, respectively (Figure 3). The bioaccessibility of EGCG in WPI- and NaCas-stabilized emulsions was significantly higher (*p* < 0.05) than that of unencapsulated EGCG solutions. However, after the gastric and intestinal phases, the bioaccessibility of encapsulated EGCG increased by approximately 64% compared to free EGCG in solution, which exhibited a bioaccessibility of only 40.93% [31]. The bioaccessibility values for WPI- and NaCas-stabilized emulsions were 65.78 ± 2.31 and 68.24 ± 1.97%, respectively, after the gastric phase, and 78.56 ± 2.14 and 81.37 ± 1.85%, respectively, following the intestinal phase. The enhanced bioaccessibility of EGCG in WPI- and NaCas-stabilized emulsions after simulated gastrointestinal digestion highlights the protective effect of the emulsion matrix against the harsh conditions encountered in the gastric and intestinal environments. The milk proteins used as emulsifiers form a robust interfacial layer surrounding the EGCG-loaded lipid droplets, providing steric and electrostatic stabilization [32]. This multilayered structure withstands the low pH and enzymatic activity in the gastric phase and enables controlled release in the intestinal phase, minimizing EGCG exposure to degradative factors. The bioaccessibility values obtained in this study are notably higher than those reported in previous investigations using different emulsion formulations. For instance, Peng et al. (2018) [11] found that the bioaccessibility of EGCG in Tween 80-stabilized nanoemulsions after simulated digestion was only 48.9%, while ref. [33] observed EGCG bioaccessibility of less than 20% in soy protein-stabilized nanoemulsions. The superior performance of WPI- and NaCas-stabilized emulsions in enhancing EGCG bioaccessibility can be attributed to the unique properties of the milk proteins, which provide a better protection and controlled release of EGCG during digestion.

### 2.4. The Anti-Proliferative Activity of EGCG

**Figure 4:**
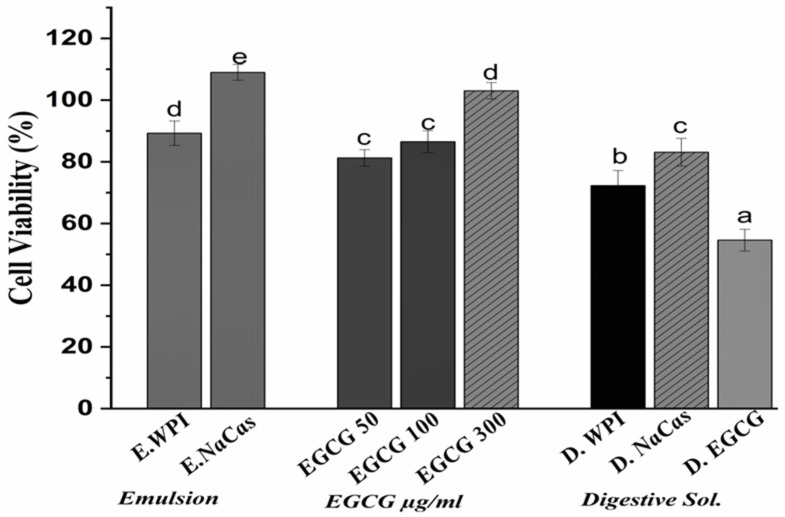
Caco-2 cell viability following a 24-h incubation period with EGCG solutions (50, 100, and 300 µg), emulsions, and digestive solution. The values represent the standard deviation ± mean of three studies. Significant differences at *p* < 0.05 are indicated by different letters.

The viability of Caco-2 cells following a 24-h incubation period with EGCG solutions (50, 100, and 300 µg/mL), emulsions, and digestive solutions is presented in Figure 4. EGCG solutions and emulsions displayed concentration-dependent anti-proliferative effects on Caco-2 cells. For the unencapsulated EGCG solution treatment, at the highest tested concentration of 300 μg/mL, cell viability was reduced to 54.6 ± 3.53%, while WPI- and NaCas-stabilized emulsions decreased cell viability to 89.11 ± 2.27 and 108.97 ± 2.5%, respectively. Digestive solutions of EGCG exhibited a more pronounced anti-proliferative effect, with cell viability dropping to 45.8 ± 2.92% at the 300 µg/mL concentration.

The results demonstrate the anti-proliferative activity of EGCG against Caco-2 colon cancer cells, agreeing with previous studies reporting the cytotoxic effects of EGCG on various cancer cell lines [34,35]. The concentration-dependent decrease in cell viability observed with EGCG solutions and emulsions highlights the potential of EGCG as a chemo-preventive agent. Interestingly, the anti-proliferative effects of EGCG were more pronounced when delivered in digestive solutions compared to undigested solutions or emulsions. This finding suggests that the simulated gastrointestinal digestion process may have enhanced the bioavailability and cellular uptake of EGCG, possibly due to the release of EGCG from the emulsion matrix and the formation of EGCG-bile salt complexes [3]. The increased bioavailability of EGCG in digestive solutions may have contributed to its more potent anti-proliferative activity against Caco-2 cells. The lower cytotoxicity observed with WPI- and NaCas-stabilized emulsions compared to EGCG solutions could be attributed to the controlled release of EGCG from the emulsion matrix, which may have modulated its cellular uptake and anti-proliferative effects. The slightly higher cell viability observed with NaCas-stabilized emulsions compared to WPI-stabilized emulsions may be due to the differences in the interactions between EGCG and the milk proteins, as well as the emulsion droplet size and stability [36]. The anti-proliferative activity of EGCG against Caco-2 colon cancer cells was demonstrated, with digestive solutions exhibiting the most potent effects. The encapsulation of EGCG in WPI- and NaCas-stabilized emulsions modulated its cytotoxicity, highlighting the potential of these delivery systems for controlled release and targeted delivery of EGCG in the gastrointestinal tract [37].

### 2.5. Oxidative Stress In Vitro by Caco-2 Cells

The present study investigated the effects of EGCG solutions, emulsions, and digestive solutions on oxidative stress in Caco-2 cells. Cell viability and intracellular ROS levels were assessed to evaluate the protective effects of the tested samples against H_2_O_2_-induced oxidative damage.

**Figure 5:**
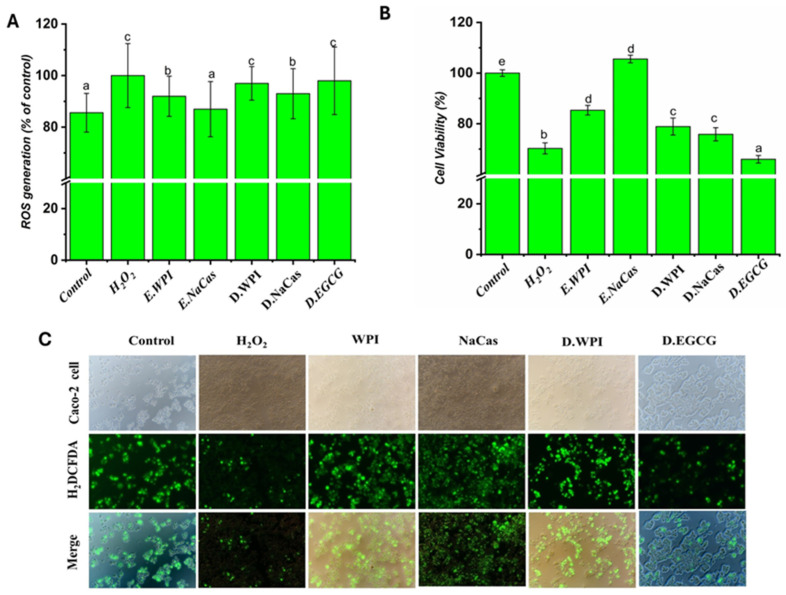
Oxidative stress in vitro by Caco-2 cells: (**A**) the level of ROS generation; (**B**) percentage of Caco-2 cell viability; (**C**) fluorescent images of H2DCFDA-stained Caco-2 cells of H_2_O_2_, EGCG solutions, emulsions, and digestive solution. Different letters denote significant difference (*p* < 0.05).

As shown in Figure 5A, H_2_O_2_ treatment significantly reduced Caco-2 cell viability to 70.28% compared to the control (*p* < 0.05), confirming its role as an inducer of oxidative stress. Among the tested samples, the NaCas emulsion demonstrated the highest cell viability at 105.56%, surpassing even the control group. This finding suggests that the NaCas emulsion not only protects cells from oxidative damage but also promotes cell growth and survival. The WPI emulsion also exhibited a protective effect, with a cell viability of 85.35%. Digestive solutions of WPI, NaCas, and EG showed lower cell viabilities of 78.90, 75.85, and 65.98%, respectively, indicating that the digestion process may alter the protective properties of the samples.

The assessment of intracellular ROS levels, as presented in Figure 5B, revealed that H_2_O_2_ treatment significantly increased ROS generation to 100.00% compared to the control at 85.60% (*p* < 0.05). This observation aligns with the well-established role of H_2_O_2_ in inducing oxidative stress [38]. The WPI and NaCas emulsions demonstrated lower ROS levels of 91.97 and 86.97%, respectively, compared to the H_2_O_2_-treated cells. These results suggest that the emulsions, particularly the NaCas emulsion, can scavenge ROS and protect cells from oxidative damage. The antioxidant properties of NaCas have been previously reported, and its ability to chelate metal ions and donate electrons contributes to its ROS-scavenging capacity [39]. Interestingly, the digestive solutions of WPI, NaCas, and EGCG showed slightly higher ROS levels of 96.98, 92.97, and 97.98%, respectively, compared to their corresponding emulsions. This observation may be attributed to the complex matrix of the digestive solutions, which could influence the stability and antioxidant activity of EGCG [39,40]. The digestive process may lead to the degradation or transformation of EGCG, resulting in a reduction of its ROS-scavenging ability [41]. Nevertheless, the digestive solutions still exhibited lower ROS levels compared to the H_2_O_2_-treated cells, indicating that EGCG retains some antioxidant properties even after digestion.

The fluorescent images of H2DCFDA-stained Caco-2 cells provide visual evidence supporting the quantitative ROS data (Figure 5C). The intense green fluorescence observed in H_2_O_2_-treated cells confirms the high level of intracellular ROS, while the reduced fluorescence intensity in cells treated with EGCG solutions, emulsions, and digestive solutions indicates their antioxidant effects. These findings are consistent with previous studies that have demonstrated the protective effects of EGCG against oxidative stress in various cell models [42].

The superior performance of NaCas and WPI emulsion in reducing oxidative stress and promoting cell viability can be attributed to several factors. First, the emulsification process may enhance the stability and bioavailability of EGCG, allowing for better cellular uptake and antioxidant activity [43]. Second, the ability of NaCas to form stable emulsions and protect EGCG from degradation during digestion may contribute to its superior performance [44].

### 2.6. Transport Study on Caco-2 Monolayer

**Figure 6:**
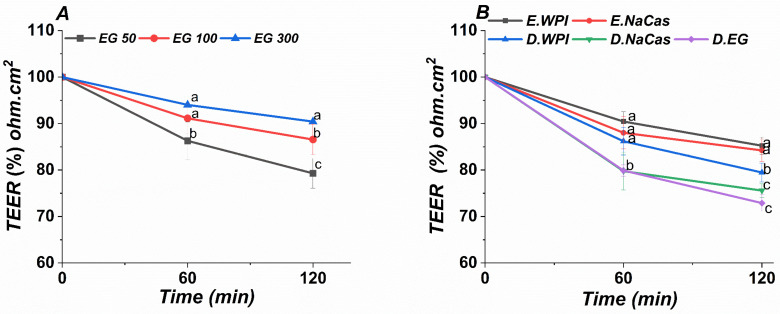
Transepithelial electric resistance (TEER) of (**A**) EGCG solutions (50, 100, and 300 µg), (**B**) WIP, NaCas emulsions, and digestive solution. The values represent the standard deviation ± mean of three studies. Different characters each time represent significant differences.

Intestinal absorption of EGCG and related polyphenols is often examined using Caco-2 monolayers as an in vitro model of the intestinal epithelium [45,46]. To investigate the potential for emulsion-based carriers to enhance EGCG delivery to intestinal cells, transport of EGCG from emulsions and solutions across Caco-2 monolayers was analyzed (Figure 6). Digestate dilutions from simulated gastrointestinal digestion were applied apically to Caco-2 layers at 50–300 μg/mL EGCG. Initial transepithelial electrical resistance exceeded 600 Ω∙cm^2^. Minimal TEER changes after 2 h incubation with emulsions and digestive solutions indicate preserved monolayer integrity across treatments. Substantial EGCG transport to the basolateral chamber was observed, suggesting the presence of intact EGCG and/or metabolites, aligning with evidence of delayed EGCG release from milk protein complexes [9]. Two experiments were conducted to evaluate the potential effects of EGCG and EGCG-loaded emulsions on Caco-2 barrier function over a 2 h period. The first experiment involved apical stimulation of Caco-2 monolayers with EGCG concentrations ranging from 50–300 μg/mL. The second experiment tested dilutions of 1:20 *v*/*v* emulsion to digestate ratios on Caco-2 cells, based on initial cytocompatibility screening.

A comparable negligible TEER reduction (~85–90% of baseline) was observed at 60 and 120 min in both studies, implying limited effects of EGCG or emulsions on tight junctions. The mild TEER decrease may reflect some tight junction modulation [47]. Since emulsions did not further reduce TEER versus EGCG alone, emulsifiers likely did not irreversibly disrupt the epithelium. Overall, the in vitro Caco-2 model demonstrates the potential of milk protein-stabilized emulsions to enable EGCG transport without compromising intestinal barrier integrity.

The Caco-2 monolayer model provides insight into the intestinal absorption and permeability potential of compounds in vitro. The results demonstrate the ability of WPI and NaCas-stabilized emulsions to facilitate the transport of encapsulated EGCG across Caco-2 cell monolayers without disrupting tight junction integrity or barrier function. Preservation of high initial transepithelial electrical resistance values despite incubation with emulsions and EGCG implies limited adverse impacts on the intestinal epithelium model. EGCG encapsulated in emulsions may avoid degradation in the harsh gastrointestinal environment, enabling more intact EGCG to reach the intestinal mucosa and become available for epithelial absorption.

### 2.7. The Apparent Permeability Coefficient (P_app_)

The *P*_app_ values for EGCG without encapsulation and in WPI and NaCas emulsions across the Caco-2 monolayer are tabulated in Table 2. *P*_app_ values were measured for EGCG encapsulated in WPI and NaCas emulsions, along with unencapsulated EGCG, using Caco-2 monolayers. *P*_app_ suggests the potential for intestinal absorption of compounds [48]. The *P*_app_ values obtained for EGCG in all forms were relatively low, suggesting limited epithelial transport. However, encapsulation of EGCG in WPI and NaCas emulsions significantly enhanced the *P*_app_ compared to unencapsulated EGCG, as shown in Table 1. The increased permeability coefficient of encapsulated EGCG indicates the emulsions facilitated paracellular and transmembrane transport across the intestinal epithelium. Protection of EGCG from degradation by the emulsions likely enabled more intact EGCG to reach the basal side of the Caco-2 monolayers The *P*_app_ values were 2.4 × 10^−6^ cm/s for unencapsulated EGCG, 3.5 × 10^−6^ cm/s for WPI emulsion, and 4.81 × 10^−6^ cm/s for NaCas emulsion. The increased permeability coefficient of encapsulated EGCG indicates the emulsions facilitated paracellular and transmembrane transport across the intestinal epithelium. Protection of EGCG from degradation by the emulsions likely enabled more intact EGCG to reach the basal side of the Caco-2 monolayers. Huang et al., 2018, demonstrated that epigallocatechin gallate (EGCG) inhibits the function of the apical sodium-dependent bile salt transporter (ASBT) in vitro in cell lines, suggesting a potential mechanism by which EGCG may decrease bile acid reabsorption in the intestine [49]. Furthermore, previous studies have shown that EGCG can bind to bile acids and alter the structure of mixed micelles composed of bile salts, phospholipids, and cholesterol, thereby reducing cholesterol solubility and potentially impacting lipid absorption. These findings provide insights into how EGCG-bile salt interactions may influence the bioavailability and cellular uptake of EGCG, as well as its effects on lipid metabolism [50,51]. These in vitro results suggest encapsulation of EGCG in WPI- and NaCas-stabilized emulsions can improve its intestinal absorption and bioavailability when administered orally [48]. Further studies are needed to confirm the metabolic fate of encapsulated EGCG. Unencapsulated EGCG undergoes extensive phase II metabolism into sulfated, glucuronidated and methylated forms when absorbed enterocytically [52]. The influence of encapsulation on the metabolic profile of EGCG will provide insight into the bioactivity of encapsulated EGCG. In vivo studies are also required to substantiate the permeability enhancement effects observed in the Caco-2 model. The digestive solutions of WPI and NaCas emulsions also exhibited higher *P*_app_ values of EGCG compared to the EGCG solutions, indicating that the encapsulation of EGCG in the emulsions improved its intestinal permeability even after the digestion process. However, the *P*_app_ values of EGCG from the digestive solutions were lower than those from the corresponding intact emulsions, suggesting that the digestion process may have affected the stability and release of EGCG from the emulsions.

## 3. Materials and Methods

### 3.1. Materials

Whey protein isolate (WPI, 80% dry base), lecithin (90% dry base), pepsin, and porcine bile salt were procured from Shanghai Yuanye Bio-Technology Co., Ltd. (Shanghai, China). The Epigallocatechin-3-gallate (EGCG, concentration > 90%) was obtained from Sigma Aldrich (St. Louis, MO, USA). Pectin with a galacturonic acid content exceeding 74.0% (dry base) was obtained from Aladdin Co., (Shanghai, China). Calcium chloride (CaCl_2_) and Span 80^®^ (sorbitane monooleate) were purchased from Sinopharm Chemical Reagent Co., Ltd., (Shanghai, China). Sodium caseinate, 3-(4,5-dimethylthiazol-2-yl)-2,5-diphenyltetrazolium bromide (MTT), dimethyl sulfoxide (DMSO), and pancreatic enzymes were acquired from Sigma Aldrich, (St. Louis, MO, USA). Caco-2 cells were procured from the Chinese Academy of Sciences’ Type Culture Collection cell bank located in Shanghai, China. Dulbecco’s Modified Eagle Medium (DMEM) and penicillin/streptomycin solution were purchased from Gibco (Beijing, China), while fetal bovine serum (FBS) was procured from Hyclone (Logan, UT, USA). The 2′,7′-dichlorofluorescin diacetate (H2DCFDA) used was acquired from Bio Frox (Shanghai, China). All other chemicals and reagents used in this study were of analytical grade. MillQ-H_2_O (Millipore, Bedford, MA, USA) was utilized in the current study.

### 3.2. Preparation of EGCG-Loaded S/O/W Emulsions and Solutions

First, aqueous solutions containing either 1% (*w*/*v*) whey protein isolate (WPI) or 0.5% (*w*/*v*) sodium caseinate (NaCas) were prepared to form the water (W) phase, according to Zhang (2015), Sabouri and Corredig (2016) and Xi (2020) [53,54,55]. Lecithin (1.0 wt.%) was incorporated into the protein solutions as an emulsifier. The mixtures were magnetically stirred for 2 h and then stored overnight at 4 °C to ensure complete hydration of the proteins. To create the solid-in-oil (S/O) phase, 0.5% (*w*/*v*) EGCG was suspended in a mixture of 90% (*w*/*v*) soybean oil and 10.0% (*w*/*w*) sorbitan monooleate (Span 80) as a surfactant. The S/O mixture was homogenized using a Cyclone I.Q. microprocessor homogenizer at 12,000 rpm for 2 min to form an initial emulsion. The S/O emulsion was then gradually added to the W phase under high-speed homogenization at 10,000 rpm for 5 min using a high-shear mixer (IKA^®^ T25 Digital Ultra-Turrax^®^, IKA, Staufen, Germany) to form a coarse S/O/W emulsion. To further reduce the droplet size and improve emulsion stability, the coarse emulsion was subjected to ultrasonication using a probe system (Scientz-IID, Ningbo Xinzhi Biotechnology Co., Ltd., Ningbo, China) with a frequency of 20 kHz and an amplitude of 40%. The emulsion was processed in an ice water bath to prevent thermal denaturation of the WPI or NaCas proteins during sonication. The ultrasonication was performed in 6 min intervals, with a 5 s on-time and a 10 s off-time for each interval. The total ultrasonication time was 30 min. The emulsification parameters were selected based on previous studies investigating the ultrasonication of similar emulsion systems [56]. The final S/O/W emulsions were stored at 4 °C until further analysis. For comparison, EGCG solutions were prepared by dissolving EGCG directly in deionized water at the same concentration (0.5% *w*/*v*) as in the emulsions.

### 3.3. Quantification of EGCG Content

The concentration of epigallocatechin gallate (EGCG) was determined through ultra-performance liquid chromatography (UPLC) coupled with a photodiode array detector (PDA). Chromatographic separation was achieved on a reverse-phase C18 column (2.1 × 100 mm, 1.7 μm particle size) maintained at a temperature of 35 °C. The mobile phase consisted of (A) water containing 0.1% formic acid and (B) methanol containing 0.1% formic acid, with a flow rate of 0.42 mL/min. The elution gradient was programmed as follows: 2% B from 0–3 min, 65% B from 3–4 min, 95% B from 4–5 min, and 2% B from 5–6 min. The injection volume was 5 μL, and the detection wavelength was set at 280 nm [57]. The EGCG concentration in the samples was quantified by constructing an external calibration curve using authentic EGCG standards ranging from 10–150 μg/mL.

### 3.4. In Vitro Gastrointestinal Digestion and Bioaccessibility Assessment

The bioaccessibility of EGCG was evaluated using a three-stage in vitro digestion model simulating oral, gastric, and small intestinal conditions, adapted from the INFOGEST consensus protocol [17] with slight modifications. Briefly, simulated salivary fluid (SSF) containing mucin and electrolytes was mixed with the samples at a 1:1 ratio and incubated at 37 °C for 2 min to mimic the oral digestion. The oral digesta were then combined with simulated gastric fluid (SGF) containing pepsin and acidified to pH 2.0 at a 1:1 ratio and incubated at 37 °C for 2 h under continuous agitation to simulate gastric digestion. Finally, simulated intestinal fluid (SIF) containing pancreatin, bile salts, and electrolytes (pH 7.0) was added to the gastric digesta and incubated at 37 °C for an additional 2 h to represent small intestinal digestion. After each digestion stage, aliquots were collected and immediately cooled to halt enzymatic reactions. For bioaccessibility, cell viability, and uptake assays, the cooled digesta were diluted 1:6 with DMEM containing 10% FBS and stored at −20 °C until further analysis [36]. For EGCG quantification, the digesta were centrifuged at 12,000 rpm for 15 min, and the supernatant (bioaccessible fraction) was filtered before UPLC analysis, as described in Section 3.3. The bioaccessibility of EGCG was calculated using the following equation:

(1) $$Bioacceessibilty\left(\%\right)=\frac{C\;in\times df}{C\;dig}\times 100$$

where C dig is the EGCG content (µg/mL) after each digestion phase, C in is the concentrations of EGCG before digestion (µg/mL) and df is the dilution factor.

### 3.5. Evaluation of EGCG Retention and Encapsulation Efficiency

The stability of EGCG-loaded emulsions was assessed by determining the retention efficiency (RE) and encapsulation efficiency (EE) during storage at 4 °C for 14 d. The RE was calculated by measuring the EGCG content in the emulsions on day 0 (initial) and after 7 and 14 days of storage using UPLC, as described in Section 3.3. The EE was indirectly determined by quantifying the free EGCG in the aqueous phase (prepared with Milli Q-H_2_O) of the emulsions. The EE and RE were calculated using the following equations:

(2) $$RE\left(\%\right)=\frac{{(M}_{storage})\;amount\;of\;EGCG\;after\;storage\;periods}{\left(M_{initial}\right)\;amount\;of\;EGCG\;in\;fresh\;samples}\times 100$$

(3) $$EE\left(\%\right)=1-\frac{M.\;Free}{M.\;Total}\times 100$$

where M. Free is the mass of free EGCG in the aqueous phase, M. Total is the total mass of EGCG added during emulsion preparation, M_storage_ is the mass of EGCG retained in the emulsions after storage, and M_initial_ is the initial mass of EGCG in the freshly prepared emulsions.

### 3.6. Molecular Docking and Dynamic Simulation

The interactions between the binding of whey protein isolate (WPI) or sodium caseinate (NaCas) and the compound epigallocatechin gallate (EGCG) were examined through molecular docking techniques. The Discovery Studio software (version 2.5), which utilizes the libdock algorithm developed by Accelrys Software Inc. (San Diego, CA, USA), was employed for this purpose. The three-dimensional structure of the major whey protein β-lactoglobulin (β-Lg, PDB code: 3NPO) was retrieved from the RCSB Protein Data Bank (http://www.rcsb.org/pdb) accessed on 10 February 2024. Whey proteins comprise approximately 18–20% of total milk proteins, and within the whey protein fraction, β-Lg is the most abundant component, representing around 65% of the whey fraction [29,58]. However, since the crystal structure of NaCas is not available in the protein database, a model of its structure was generated using the I-TASSER server [59,60]. The compound EGCG (PubChem CID: 65064) was used as the ligand molecule. The protein structures were prepared by removing water molecules and adding hydrogen atoms. Prior to docking, potential binding pockets on the proteins were identified using site finding tools to locate concave regions in their three-dimensional structures. The search for binding sites was initiated from cavities within the protein structures. Energy minimization was carried out using the BEST protocol, which involves the best-Newton minimization in Cartesian space, conjugate-gradient minimization in torsion space, and conjugate-gradient minimization. The docking procedure itself was performed using the libdock method, which treats the proteins as rigid bodies and allows full flexibility for the small ligand molecule. The docked model with the highest score (lowest docking energy) was selected to represent the most favorable binding mode. Molecular dynamics (MD) simulations were conducted for a total of 80 nanoseconds, with snapshots saved every 10 nanoseconds. The geometry of EGCG was optimized using the M062X functional with the 6-31G (d, p) basis set, aided by the Gaussian 09 package. Atomic charges were reassigned using the RESP algorithm, and solvation effects were accounted for using the IEF-PCM model. EGCG was randomly placed around β-Lg or NaCas (within a 20 Å radius), subjected to double minimization, and the docking was performed [1,60].

### 3.7. Cell Culture

The human colorectal adenocarcinoma cell line Caco-2 was utilized for in vitro experiments. Caco-2 cells were cultured in Dulbecco’s Modified Eagle’s Medium (DMEM) supplemented with 10% fetal bovine serum, 100 U/mL penicillin/streptomycin antibiotics, and 1% non-essential amino acids. The cells were incubated at 37 °C with 5% CO_2_ under humidified conditions. Passages between 10 and 25 were employed for all experiments.

### 3.8. Assessment of the Cell Viability

The anti-proliferative effects of EGCG delivered in emulsions compared to solutions before and after in vitro digestion were evaluated using an MTT colorimetric cytotoxicity assay in Caco-2 cells.

Caco-2 cells were seeded at a density of 1 × 10^4^ cells per well in 96-well plates and allowed to grow for 24 h before treatment, as described by [61]. Subsequently, the cells were incubated with 150 μL of emulsions, digested emulsions, or EGCG solutions at a 1:20 dilution in culture medium for 24 h. After the treatment period, MTT was added to a final concentration of 0.5 mg/mL (150 μL/well) and incubated for 4 h. The formazan crystals produced by viable cells were solubilized in 150 μL of dimethyl sulfoxide (DMSO) per well. Absorbance was measured at a wavelength of 570 nm using a Cytation 3 multifunctional microplate reader (BioTek Instruments, Inc, Winooski, VT, USA). Cell viability was calculated according to the following equation:

(4) $$\mathrm{Cell}\;\mathrm{viability}\;(\%)=\frac{\left(A_{test}-A_{blank}\right)}{\left(A_{control}-A_{blank})\times 100\;(\%\right)}$$

where A_test_ and A_control_ are the absorbance values of the cells with and without dosing, respectively, and A_blank_ is the absorbance value of DMSO.

### 3.9. Transport Study with Caco-2 Confluent Monolayer and Bidirectional Permeability

The transport study was conducted using Caco-2 cell monolayers to evaluate the permeability and transport efficiency of EGCG from various formulations, including solutions, emulsions, and their digestive products. Caco-2 cells were seeded onto polycarbonate membrane inserts (0.4 μm pore size, 1.12 cm^2^ growth area) in 12-well plates at a density of 1 × 10^5^ cells/cm^2^. The cells were cultured in DMEM supplemented with 10% FBS, 1% non-essential amino acids, 100 U/mL penicillin, and 100 μg/mL streptomycin at 37 °C in a humidified atmosphere with 5% CO_2_. The culture medium was replaced every other day, and the cells were used for experiments 21 d post-seeding. The integrity of the Caco-2 cell monolayers was evaluated by measuring the transepithelial electrical resistance (TEER) using a Millicell-ERS-2 volt-ohm meter (Millipore, USA). The TEER values were calculated as a percentage of the initial value using the following equation:

(5) $$\mathrm{TEER}\;(\%)=\frac{Rt}{R0}\times 100$$

where Rt is the resistance at time t, and R0 is the initial resistance. Monolayers with initial TEER values above 500 Ω·cm^2^ were used for transport experiments (Zhang et al., 2004) [62].

After 21 d, EGCG solutions (50, 100, and 300 μg/mL), WPI and NaCas emulsions, and their digestive solutions (0.5 mL) were added to the apical side of the Caco-2 cell monolayers. The basolateral side was filled with 1.5 mL of Hank’s balanced salt solution (HBSS). The plates were incubated at 37 °C with 5% CO_2_. TEER measurements were performed at 0, 60, and 120 min.

The basolateral solutions were collected after 2 h of incubation with treatments and acidified to pH 2.5 using 1% ascorbic acid with 0.28% phosphoric acid before freeze-drying overnight and storage at −20 °C until further processing. Using ascorbic acid protects tea polyphenol and catechins from oxidative and degradation [45]. UPLC analysis was used to quantify transported EGCG levels as previously described in Section 3.3.

The apparent permeability coefficient (*P*_app_) of EGCG and the transport rate was expressed in μg/sec/cm^2^ which was calculated using the following equation:

(6) $$P_{\mathrm{app}}=\frac{\left(\Delta Q\right)}{\left(\Delta ts\right)}\times \frac{1}{\left(A\times C0\right)}$$

where: (ΔQ) is the amount of EGCG appearing in the receiver side (μg), (Δt_s_) the duration (s), (Δ t_m_) is the duration (min), (A) surface area of the membrane (cm^2^), and (C_0_) is initial amount in the donor chamber (µg) (Song et al., 2014) [63].

### 3.10. Assessment of Oxidative Stress in Caco-2 Cell Model

The cytoprotective effect of EGCG-loaded WPI and NaCas emulsions against hydrogen peroxide (H_2_O_2_)-induced oxidative damage was evaluated using the human colorectal adenocarcinoma Caco-2 cell line. Cells were seeded in 96-well plates at a density of 1 × 10^4^ cells per well and cultured for 24 h prior to treatment. To induce oxidative stress, cells were exposed to 1 mM H_2_O_2_ for 4 h. Subsequently, cells were treated with different concentrations (50, 100, and 300 μg/mL) of free EGCG or EGCG-loaded emulsions for an additional 24 h. Cell viability was assessed using the MTT colorimetric assay as described in Section 3.8. Intracellular reactive oxygen species (ROS) levels were quantified using the fluorescent probe H2DCFDA. A microplate reader (Tecan Infinite 200 pro, Männedorf, Switzerland) was used to measure the intensity of the fluorescence at 485 nm for excitation and 530 nm for emission. The ROS levels were measured with the following formula [64]:

(7) $$ROS\left(\%\right)\frac{\left(F.\;Sample-F.\;Control\right)}{\left(F.\;H_{2}O_{2}-F.\;Control\right)}\times 100$$

where F. Sample, F. Control, and F. H_2_O_2_ represent the fluorescence intensities of the sample, control, and H_2_O_2_-treated cells, respectively. Cell viability was determined using the MTT assay as described in Section 3.8. The fluorescent images were obtained for each well using a Nikon Ti–S fluorescent microscope (Nikon, Tokyo, Japan).

### 3.11. Statistical Analysis

All experiments were conducted in triplicate and results were expressed as mean ± standard deviation. Data were analyzed by one-way ANOVA followed by Duncan’s post-hoc test at *p* < 0.05 using SPSS Statistics V27.0 to determine significant differences among means.

### 3.12. Experimental Overall Flow Chart

**Figure 7:**
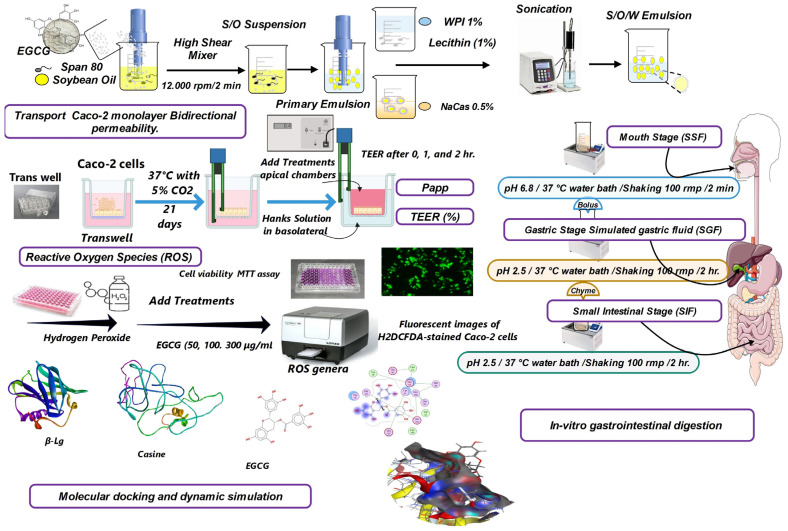
Overall experimental design flow chart.

The overall experimental workflow is summarized in Figure 7, depicting the key steps of emulsion preparation, characterization, in vitro digestion, cell culture studies, and computational modeling employed in this investigation.

## 4. Conclusions

Herein, we investigated the potential of WPI and NaCas emulsions as delivery systems to enhance the intestinal permeability and absorption of EGCG using a Caco-2 cell monolayer model. The results demonstrated that encapsulation of EGCG in WPI and NaCas emulsions significantly improved its transport and permeability across Caco-2 cell monolayers compared to EGCG solutions. Molecular docking and dynamics simulations provided valuable insights into the binding interactions between EGCG and the biological macromolecules, further elucidating the mechanisms behind their encapsulation capabilities and protective effects. The protective effect of the emulsions on EGCG was evident from the higher apparent permeability observed for the emulsion-based delivery systems. Furthermore, NaCas-emulsions exhibited superior performance in enhancing the intestinal transport and permeability of EGCG compared to WPI-emulsions, which can be attributed to the excellent emulsifying properties of NaCas and its ability to form stable complexes with EGCG. The digestive solutions of both emulsions also showed higher apparent permeability values compared to EGCG solutions, indicating that the encapsulation maintained a protective effect after digestion. These findings contribute to the growing the research on the development of innovative delivery systems for dietary supplements and bioactive compounds, aligning with the broader trend in the field that focuses on improving the delivery and absorption of these compounds to maximize their health benefits. The use of milk proteins as emulsifiers offers a promising approach to enhance the stability, bioavailability, and efficacy of polyphenols like EGCG. However, future research should investigate the performance of these emulsion-based delivery systems in animal models and human clinical trials to validate their efficacy and safety.

## Figures and Tables

**Table 1:** Binding energies and amino acids are involved in these interactions between β-lactoglobulin, sodium caseinate, and EGCG, as predicted by molecular docking simulations.

Receptor	Binding Energy (kcal/mol)	The Amino Acids Involved in These Interactions	Binding Pose
β-lactoglobulin	−5.035	Glu91, Thr120, Leu151, Glu36, Glu37, Pro90, Gln34, Phe33, Met93, LysA32, Val116, and Thr120.	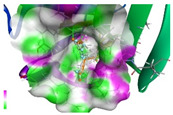
NaCas	−5.979	Glu45, 44, 157, 158, leu57,56, Gln68, 159, 59, Tyr20, Val43, His161, and Thr18.	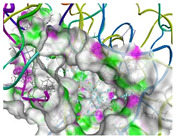

**Table 2:** *P*_app_ values for emulsions and EGCG solution, loading in the apical and basolateral compartment of Caco-2 monolayer.

Emulsions and Solution	Initial	Applied in Apical Compartment	Applied in Basolateral Compartment	Apparent Permeability Coefficient (cm/s)
	Concentration of EGCG (µg/mL)			*P*_app_ × 10^−6^ ± SD
WPI	290.14 ± 1.49	32.94 ± 3.42	32.01 ± 0.56	3.5 ± 0.33
NaCas	194.55 ± 1.68	31.94 ± 1.94	30.702 ± 1.58	4.81 ± 1.02
EG Solutions	55.79 ± 2.01	30.87 ± 1.14	29.013 ± 0.52	2.461.42

The values represent the averages of three separate studies with a standard deviation.

## Data Availability

The data presented in this study are available in article and Supplementary Materials.

## Supplementary Materials

The following supporting information can be downloaded at: https://www.mdpi.com/article/10.3390/molecules29112588/s1, Table S1. The docking parameters of the binding between EGCG with NaCas or β-Lg.
