# Supplementary material for: Whey Protein Sodium-Caseinate as a Deliverable Vector for EGCG: In Vitro Optimization of Its Bioaccessibility, Bioavailability, and Bioactivity Mode of Actions
Source: Molecules. 2024 May 31;29(11):2588. doi: 10.3390/molecules29112588 (PMC11174060; doi:10.3390/molecules29112588)
Supplement: Supplementary file 1 [file molecules-29-02588-s001.zip › molecules-3027079-supplementary.pdf]

Table S1. The docking parameters of the binding between EGCG with NaCas or β-Lg.

NaCas-EGCG

	mol	rseq	mseq	S	rmsd_refine	E_conf	E_place	E_score1	E_refine	E_score2
1	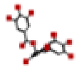	1	1	-7.8483	1.1111	-26.3084	-95.0688	-13.6267	-36.7951	-7.8483
2	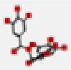	1	1	-7.3711	1.4928	-26.7983	-110.3496	-14.7116	-38.5098	-7.3711
3	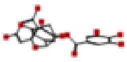	1	1	-6.8579	2.1078	-27.0464	-101.6240	-13.6326	-27.6974	-6.8579
4	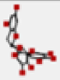	1	1	-6.5413	1.2484	-26.3812	-78.7930	-14.1624	-28.7779	-6.5413
5	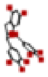	1	1	-6.3410	3.0586	-29.6232	-88.0868	-14.2313	-27.3550	-6.3410

β-Lg-EGCG

	mol	rseq	mseq	S	rmsd_refine	E_conf	E_place	E_score1	E_refine	E_score2
1	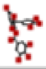	1	1	-6.3294	1.6178	-30.4630	-42.5146	-14.0509	-32.5518	-6.3294
2	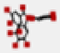	1	1	-5.8763	1.2524	-30.7017	-70.4946	-13.6895	-34.4661	-5.8763
3	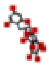	1	1	-5.5901	1.7197	-32.3823	-78.2201	-13.2705	-29.6301	-5.5901
4	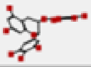	1	1	-5.5753	1.6279	-33.8848	-78.9955	-14.3047	-29.7971	-5.5753
5	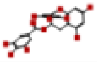	1	1	-5.2432	0.9913	-32.0115	-94.9043	-13.2556	-33.8476	-5.2432
